# Clinical features of autonomic dysfunction in children with anti-N-methyl-D aspartic receptor encephalitis

**DOI:** 10.1186/s13052-025-01857-4

**Published:** 2025-01-18

**Authors:** Dongqing Li, Jing Sun, Guannan Li, Shuo Miao, Jian Yang, Jianzhao Zhang

**Affiliations:** Neurology Department of Children’s hospital affiliated to the Capital Institute of Pediatrics, Beijing, China

**Keywords:** Anti-NMDAR encephalitis, Autonomic dysfunction, Children, Encephalitis

## Abstract

**Background:**

Anti-N-methyl-D-aspartic receptor encephalitis (Anti-NMDAR encephalitis) is the most prevalent form of autoimmune encephalitis in pediatric patients. Autonomic dysfunction is a frequent symptom of Anti-NMDAR encephalitis, yet it often goes unnoticed by pediatricians. Studies have indicated that pediatric patients with autonomic dysfunction exhibit a poorer prognosis compared to those without. To date, research on autonomic dysfunction in encephalitis has predominantly focused on adults, with no studies conducted on pediatric populations. This analysis examines the clinical features of pediatric patients with Anti-NMDAR encephalitis complicated by autonomic dysfunction.

**Methods:**

We performed a retrospective analysis of patients diagnosed with Anti-NMDAR encephalitis at the Department of Neurology, Children’s Hospital affiliated to the Capital Institute of Pediatrics, from June 2017 to June 2023. Patients were categorized based on the presence or absence of autonomic dysfunction during their illness. We summarized and compared the clinical features of children with autonomic dysfunction and analyzed the risk factors for its development in pediatric Anti-NMDAR encephalitis patients.

**Results:**

A total of 56 children were included in this study. Twenty-two (39.3%) exhibited autonomic nervous dysfunction. The most prevalent symptom of autonomic dysfunction was cardiovascular autonomic dysfunction(21/22, 95%),with the specific manifestations being sinus tachycardia (8 cases), ventricular premature beats (2 cases), atrioventricular block (2 cases), atrial premature beats (3 cases), and sinus bradycardia (4 cases),hypertension(1 case) and cardiac arrest(1 case). Other symptoms included gland secretion dysfunction (19/22, 86%),ventilate dysfunction(3/22,14%), thermoregulatory dysfunction (3/22,14%), bladder dysfunction(2/22,9%). Compared to the group without autonomic dysfunction, the group with dysfunction showed significantly higher rates of prodrome infection, tumor complications (all ovarian teratoma), consciousness disturbance, elevated cerebrospinal fluid protein, initiation of second-line and long-term immunotherapy, length of hospital stay, and hospitalization costs (*P* < 0.05).

**Conclusion:**

Among pediatric patients with Anti-NMDAR encephalitis, cardiovascular autonomic dysfunction is the most common form of autonomic dysfunction. Those with autonomic dysfunction have a worse prognosis and longer hospital stays. Active initiation of second-line and long-term immunotherapy is recommended.

**Supplementary Information:**

The online version contains supplementary material available at 10.1186/s13052-025-01857-4.

## Background

Anti-NMDAR encephalitis is the most common autoimmune encephalitis in children, accounting for approximately 54–80% of cases [1]. The disease presents with prodromal symptoms such as fever and headache or events of central nervous system infection. Main symptoms include abnormal mental behavior, cognitive impairment, seizures, dysphasia, dyskinesia, consciousness disturbances, and autonomic nervous dysfunction. Dysphasia can be manifested as aphasia, dysarthria, imitation speech, mustism, mumbling,etc [2]. The clinical manifestations of dyskinesia are diverse, such as dystonia, chorea, stereotypies, oral and facial involuntary movements, tremors, myoclonus, myotonia, athetosis, limb paralysis, ataxia, etc [3, 4].

Anti-NMDAR encephalitis can damage the central autonomic neural network, leading to sympathetic and parasympathetic dysfunction, resulting in autonomic dysfunction, including sinus tachycardia, sinus bradycardia, increased salivation, hypotension, central fever, and central hypoventilation [5, 6]. Autonomic dysfunction is a common clinical manifestation in anti-NMDA receptor encephalitis, which can cause substantial morbidity.Despite its frequency, autonomic nervous dysfunction in Anti-NMDAR encephalitis is often overlooked by pediatricians, leading to underestimation of associated risks. Patients with autonomic dysfunction usually have a severe initial condition and a very high mortality rate. Patients mainly die from respiratory and circulatory dysfunction and failure, and the prognosis is poor. Early recognition, aggressively cardiorespiratory support in the Intensive Care Unit and supportive care treatment of autonomic dysfunction can improve the prognosis of pediatric patients with anti-NMDA receptor encephalitis.Currently, studies on Anti-NMDAR encephalitis with autonomic dysfunction are limited to adults, with none focused on pediatric patients. This analysis aims to enhance understanding and recognition of Anti-NMDAR encephalitis with autonomic nervous dysfunction in children by investigating its clinical features and differences between groups with and without this complication.

## Methods

This retrospective study received approval from the Ethics Committee of the Capital Institute of Pediatrics (Ethics number: SHERLLM2024017), which exempted guardians from providing informed consent.All the design, analysis, interpretation of data, drafting and revisions followed the guidelines for reporting observational studies (STROBE) [7].

Sample size estimation was conducted using PS-Power Simple Size software (version 3.1.2). As no prior studies have examined the autonomic dysfunction in pediatric patients with Anti-NMDAR encephalitis, estimation of required sample size is based on our clinical practice. Our unpublished data in small samples have shown that the average level of mRS score at 3rd month was 1 in patients without autonomic dysfunction, and 3 in patients with autonomic dysfunction. After assuming two-sided α of 0.05 and power of 1 – β (0.1) at 0.9, an estimated 22 patients are required in each group. In practice, we have enrolled 34 patients without autonomic dysfunction and 22 patients with autonomic dysfunction, which met the theoretically minimal sample size requirement and renders us to have relatively sufficient power to detect statistical significance.

### Study population

This study included pediatric patients diagnosed with anti-NMDAR encephalitis at the Children’s Hospital affiliated to the Capital Institute of Pediatrics from June 2017 to June 2023. The inclusion criteria were based on the diagnostic framework proposed by Graus [5] in 2016, requiring that patients meet all three of the following conditions: A, B, and C. Condition A necessitates one or more of six main symptoms: (1) abnormal mental behavior or cognitive impairment, (2) speech disorder, (3) seizures, (4) dyskinesia/involuntary movement, (5) decreased level of consciousness, and (6) autonomic nervous dysfunction or central hypopnea. Condition B confirms Anti-NMDAR antibody positivity, with cerebrospinal fluid CBA antibody positivity being prevalent. Condition C involves the reasonable exclusion of other causes. Additionally, patients must have had a follow-up period of more than 3 months. The exclusion criterion was the presence of incomplete clinical data,including non-compliance with treatment and non-regular follow-up.

### Data collection and grouping

Data were collected on demographic details, clinical symptoms, and results of ancillary tests for patients with anti-NMDAR encephalitis. The information gathered included age, sex, prodromal symptoms, main symptoms, presence of concomitant tumors, routine biochemical and cytological tests of cerebrospinal fluid, detection of anti-NMDAR antibodies in both blood and cerebrospinal fluid, use of mechanical ventilation, results of head magnetic resonance imaging, hospitalization costs, the Glasgow Coma Score (GCS), the modified Rankin Scale (mRS) at admission, the maximum duration of disease, and the status at three months post-discharge.

Patients were categorized based on the presence or absence of autonomic dysfunction during their illness. In this study, the categorization of the symptoms of autonomic dysfunction was refered to previously published literature reports and summarized according to affected organs [5, 8]. This classification is appropriate and targeted for the study of autonomic nervous dysfunction in anti-NMDAR encephalitis, making the study results more applicable to clinical diagnosis and treatment.Autonomic dysfunction encompassed a range of symptoms including cardiovascular autonomic dysfunction (such as sinus tachycardia, sinus bradycardia, atrioventricular block, sinus atrial block, atrial premature beat, ventricular premature beat, atrial tachycardia, ventricular tachycardia hypertension,and cardiac arrest), thermoregulation dysfunction, gland secretion dysfunction, bladder voiding dysfunction and ventilation dysfunction. Conditions such as underlying diseases causing arrhythmia were excluded. Manifestations not attributable to other causes like fever, crying, or shock were considered indicative of autonomic dysfunction.

Clinical symptoms were classified into several categories: abnormality of mental behavior or cognitive impairment,dysphasia, seizures, dyskinesia/involuntary movement, consciousness disorders, and ataxia. The number of clinical symptoms per patient was tallied. The count also included patients experiencing epileptic status, defined as seizure activity lasting more than 30 min. Mechanical ventilation was noted for patients requiring ventilator assistance post-intubation or tracheotomy. The GCS was utilized to assess the state of consciousness. Treatment modalities were recorded as follows: first-line treatments included human immunoglobulin, glucocorticoids, and plasma exchange; second-line treatments covered rituximab and cyclophosphamide; and long-term immunotherapy involved agents like mycophenolate mofetil and mercaptopurine.

Follow-up: After discharge, patients were followed up regularly through outpatient or telephone visits, once a month for 3 months. Patients were hospitalized for evaluation at 3 months after discharge. The modified Rankin scale (mRS) score was used to evaluate neurological function. Experienced peadiatric neurologists record the highest mRS Score during the course of the disease, mRS Score at discharge and mRS Score at return visit 3 months after discharge.

### Statistical analysis

The data were analyzed using SPSS26.0 statistical software. Count data were represented by the number of cases and percentage (%), with comparisons made using the Chi-square test or Fisher’s exact test as appropriate. The Shapiro–Wilk test was employed to assess the normality of the measurement data. For data adhering to a normal distribution, results were expressed as mean ± standard deviation, and comparisons between groups were conducted using the independent sample T-test. Measurement data not conforming to a normal distribution were represented by the median and interquartile range (M(Q1-Q3)), with group comparisons performed using the non-parametric Mann–Whitney U rank sum test. A significance level was set at *P* < 0.05.

## Results

### Clinical manifestations and demographic characteristics of autonomic nervous dysfunction in anti-NMDAR encephalitis complicated with autonomic nervous dysfunction

**Fig. 1:**
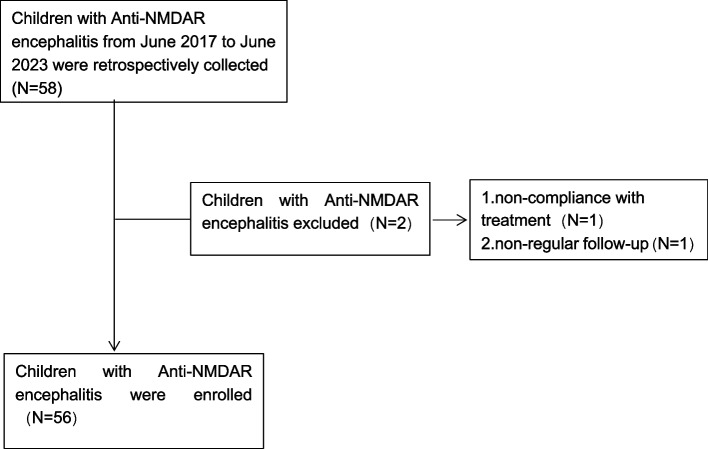
Patient inclusion/exclusion flowchart

The study included 56 children diagnosed with Anti-NMDAR encephalitis (Fig. 1), 22 of whom exhibited symptoms of autonomic dysfunction while 34 did not, resulting in an incidence rate of autonomic dysfunction of 39.3%. The onset of autonomic dysfunction ranged from the 3rd to the 21st day of the disease course, with a median onset time of 9 days. Autonomic dysfunction is simplified to cardiovascular autonomic dysfunction,thermoregulation dysfunction, gland secretion dysfunction, bladder voiding dysfunction and ventilation dysfunction.Cardiovascular autonomic dysfunction was the most frequent symptom of autonomic dysfunction (21/22), detailed as follows: sinus tachycardia in 8 patients, ventricular premature beats in 2 patients, atrioventricular block in 2 patients, atrial premature beats in 3 patients, sinus bradycardia in 4 patients,hypertension in 1 patient and cardiac arrest in 1 patient.Other symptoms included gland secretion dysfunction (19/22),ventilation dysfunction(3/22), thermoregulation dysfunction (3/22), bladder voiding dysfunction(2/22), as shown in Table 1.

**Table 1:** Clinical manifestation and incidence of autonomic nervous dysfunction

Autonomic dysfunction	patients (*n* = 22)	percent (%)
cardiovascular autonomic dysfunction	21	95
gland secretion dysfunction	19	86
ventilation dysfunction	3	14
thermoregulation dysfunction	3	14
bladder voiding dysfunction	2	9

Cardiovascular autonomic dysfunction include sinus tachycardia (*n* = 8), ventricular premature beats (*n* = 2), atrioventricular block (*n* = 2), atrial premature beats(*n* = 3), sinus bradycardia (*n* = 4),hypertension (*n* = 1),and cardiac arrest (*n* = 1).Gland secretion dysfunction include increased salivation (*n* = 16) and hyperhidrosis (*n* = 3)

### Comparison of clinical features between patients with and without autonomic dysfunction

As detailed in Table 2, there were no significant differences in sex and age between patients with and without autonomic dysfunction. In the group with autonomic dysfunction, 4 patients had tumors (all ovarian teratomas) and 14 patients exhibited prodromal symptoms. Conversely, no patients in the group without autonomic dysfunction had tumors, and 11 had prodromal symptoms. The presence of prodromal symptoms and tumors was higher in the group with autonomic dysfunction. Although the number of major symptoms was similar between the two groups, there were notable differences in the disturbance of consciousness. In the group with autonomic dysfunction, 18 patients experienced disturbances in consciousness, with a median GCS score of 9 (interquartile range: 7.75–11.25). In contrast, in the group without autonomic dysfunction, 14 patients experienced consciousness disturbances, with a median GCS score of 15 (interquartile range: 10–15)..Regarding auxiliary examinations, there were no significant differences between the two groups in cerebrospinal fluid white blood cell count, MRI abnormalities, blood anti-NMDAR antibody titer, or cerebrospinal fluid anti-NMDAR antibody titer. In terms of treatment, patients with autonomic dysfunction received first-line treatments, second-line treatments, and long-term immunotherapy. The rates of mechanical ventilation, length of hospital stay, and hospitalization costs were greater in the group with autonomic dysfunction. Furthermore, the highest mRS score, mRS score at discharge, and mRS score three months post-discharge were higher in the group with autonomic dysfunction, indicating a poorer prognosis.

**Table 2:** Comparison of clinical features between patients with and without autonomic dysfunction

	Group without autonomic dysfunction (*n* = 34)	Group with autonomic dysfunction (*n* = 22)	t/z/x^2^	*P*
Sex			0.322	0.565
Male, n(%)	15 (44.12)	8 (36.36)		
Femal, n(%)	19 (55.88)	14 (63.64)		
Age,mean ± SD, mouth	82.88±41.89	92.73 ± 43.86	−0.84	0.403
Disturbance of consciousness, n(%)	14 (41.18)	18 (81.82)	9.009	**0.003**
Duration of disturbance of consciousness (day)	0.00(0.00–20.25)	33.50(23.25–52.50)	−3.819	**< 0.001**
GCS score	15.00(11.00–15.00)	9.00(7.75–11.25)	3.566	**< 0.001**
Number of symptom types	3.00(2.00–3.00)	3.00(2.00–4.00)	−0.566	0.572
Epilepsy, n(%)	21 (61.76)	13 (59.09)	0.040	0.841
Status epilepticus, n(%)	9 (26.47)	9 (40.91)	1.277	0.259
WBC count of CSF(10^6^/L)	14.00(4.75–35.50)	17.00(4.75–52.00)	−0.260	0.794
Protein of CSF (mg/L)	188.55(132.23–267.45)	246.15(167.4–382.38)	−2.064	**0.039**
Tumour, n(%)	0 (0%)	4 (18.18)	-	**0.02**
Blood anti-NMDAR antibody titer	10.00(0.00–32.00)	10.00(0.00–32.00)	0.151	0.88
CSF anti-NMDAR antibody titer	100.00(32.00–100.00)	100.00(32.00–100.00)	0.447	0.655
mechanical ventilation, n(%)	1 (2.94)	4 (18.18)	-	0.072
MRI abnormalities, n(%)	11 (32.35)	9 (40.91)	0.426	0.514
The highest mRS score	4.00(4.00–4.25)	5.00(4.75–5.00)	−4.019	**< 0.001**
mRS score at discharge	2.00(1.00–2.25)	3.50(3.00–4.00)	−4.887	**< 0.001**
mRS score three months post-discharge	1.00(0.00–1.00)	3.50(3.00–4.00)	−4.438	**< 0.001**
length of hospital stay (day)	23.00(18.00–30.50)	35.00(25.25–42.00)	−2.742	**0.006**
Hospitalization costs (yuan)	50,544.00(31,521.50–92796.50)	126,845.00(65,096.75–229,278.75)	−3.608	**< 0.001**
Numbers of immunotherapies	2.00 (2.22–3.00)	3.00 (3.00–3.00)	−3.073	**0.002**
Prodromal symptoms, n(%)	11 (32.65)	14 (63.64)	5.29	**0.021**

*GCS *Glasgow Coma Score, *CSF* cerebrospinal flfluid, *NMDAR* N-methyl-d-aspartate receptor, *MRI* magnetic resonance imaging, *WBC* white blood cell count, *mRS* modifified Rankin scale

### Multivariate firth logistic regression analysis of the associated risk of anti-NMDAR encephalitis with autonomic dysfunction

Using the presence of autonomic nervous dysfunction as the dependent variable (0 = good prognosis, 1 = poor prognosis), a Multivariate Firth Logistic Regression analysis was conducted. The analysis included factors identified as significant in univariate analysis (history of preinfection, disturbance of consciousness, GCS score, duration of consciousness disturbance, tumor presence, highest modified mRS score, cerebrospinal fluid protein). A significant correlation was found between the highest modified mRS score and autonomic dysfunction (*P* < 0.05), as shown in Table 3.

**Table 3:** Multivariate Firth Logistic regression analysis of the associated risk of anti-NMDAR encephalitis with autonomic dysfunction

	β	SE	OR	95%CI	χ2	P
Prodromal symptoms	1.048	0.663	2.853	0.755–12.342	2.381	0.123
Disturbance of consciousness	−0.674	1.082	0.509	0.049–4.318	0.373	0.541
GCS score	−0.075	0.171	0.928	0.632–1.369	0.155	0.694
Duration of disturbance of consciousness	0.002	0.020	1.002	0.961–1.061	0.008	0.928
Tumour	1.396	1.481	4.040	0.287–584.099	0.965	0.326
highest modified mRS score	1.720	0.823	5.583	1.055–41.484	4.223	0.040
CSF protein	0.004	0.002	1.004	0.999–1.009	2.658	0.103

Results Improved mRS Score (highest) was an independent risk factor for autonomic dysfunction, with a 4.583-fold increase in risk for each 1-point increase

## Discussion

Anti-NMDAR encephalitis is the most prevalent form of autoimmune encephalitis, with autonomic dysfunction occurring in approximately 45.5% to 69% of patients [8–11]. Sinus tachycardia is the most frequent symptom of autonomic dysfunction, followed by hypertension and constipation. A minority of patients [8, 12] may also experience cardiac arrest, necessitating the use of temporary pacemakers. Symptoms of autonomic dysfunction often resolve following immunotherapy [13, 14]. Anti-NMDAR encephalitis with autonomic dysfunction is severe, characterized by a high incidence of teratomas, seizures, involuntary movements, and consciousness disorders, as well as a high rate of complications such as lung infections. The short-term prognosis for these patients is poor, and treatment typically requires the use of multiple immunosuppressants. Such patients often need more proactive admission to the intensive care unit [8, 11, 15]. However, previous studies have focused on Anti-NMDAR encephalitis in adults, there are fewer studies involving children.

The patients of anti-NMDAR at different ages will display different clinical features [9]. The most common clinical manifestations of adults with anti-NMDAR encephalitis are psychiatric features, cognitive impairment and autonomic dysfunction. However, most pediatric of anti-NMDAR encephalitis presented with psychiatric features, dyskinesia and convulsions, and they have a lower proportion of autonomic instability than adults [16, 17]. In Ying Wang’s et al. study, 23.5% of pediatric with anti-NMDAR encephalitis with demonstrated autonomic dysfunction [18], compared to 37–86% adult patients displayed autonomic symptoms [9, 17]. Although some studies have noted that pediatric patients also suffer from autonomic symptoms, but they are more concerned about other symptoms such as seizures. Previous studies have not realized the relationship between autonomic dysfunction and prognosis in pediatric patients [9, 19]. Consequently, in clinical settings, doctors and nurses often overlook symptoms of autonomic dysfunction in children, leading to delays in recognition and treatment [9, 20]. This study specifically targeted pediatric patients and found that more than one-third of children with Anti-NMDAR encephalitis experienced autonomic nervous dysfunction, highlighting that this is not an uncommon occurrence. Results from this study revealed that the most common manifestations of autonomic dysfunction were cardiovascular autonomic dysfunction, particularly tachycardia and bradycardia, followed by increased salivation, aligning with findings from previous studies. Additionally, a small number of patients also presented with severe complications such as prolonged cardiac arrest and Takotsubo cardiomyopathy, requiring the use of temporary pacemakers [7, 21]. Among the 56 children in this study, one case involved a sudden long-term cardiac arrest that resulted in death despite resuscitation efforts, while the symptoms in other children resolved following immunotherapy.

The autonomic nervous system is consisted of a complex neurons and pathways, involves central and peripheral nervous system components, wide ranging and extends to most organ systems in the body [22, 23]. The system regulate quickly the blood pressure, heart rate, temperature, vascular reactivity, bowel function, bladder function and thermoregulation in internal and external changes. The center autonomic nervous incorporate some interconnected areas distributed in the forebrain,brainstem, and spinal cord, controling the activity of preganglionic sympathetic and parasympathetic neurons [24]. The peripheral nervous system including the sympathetic, parasympathetic, and enteric nervous systems, maintain regional reflex control of autonomic function with modulating input from central autonomic systems. Norepinephrine is the primary neurotransmitter of sympathetic ganglion neurons, working on target organs by different subtypes of α1, α2, and β adrenoceptors [22]. The primary neurotransmitter in parasympathetic ganglion neurons and enteric nervous system neurons is acetylcholine, act through different subtypes of excitatory M1 receptors and M3 receptors, as well as inhibitory M2 receptors [22, 23]. For various diseases, the sympathetic and parasympathetis nerves are out of balance, resulting in autonomic dysfunction [25]. Sympathetic underactivity can triggersyncope, bradycardia and anhidrosis. Sympathetic overactivity may cause paroxysmal hypertension, extrasystoles, peripheral vasoconstriction, and hyperhidrosis [23, 26, 27]. Parasympathetic underactivity may cause tachycardia, bladder voiding and gastrointestinal dysfunction, while parasympathetic overactivity trigger paroxysmal bradycardia, asystole, fever or gastrointestinal hypermotility [23, 26, 27].

The etiology of autonomic dysfunction in patients with anti-NMDAR encephalitis remains unclear. Some studies have suggested that this dysfunction may be associated with factors such as cerebrospinal fluid glucose levels, anti-NMDAR antibody titers, types of immunotherapy, and temporal lobe lesions [12, 13]. However, Byun et al. [10]compared patients with anti-NMDAR encephalitis to those with herpes simplex encephalitis and found that autonomic nervous dysfunction in anti-NMDAR encephalitis was not related to temporal lobe lesions. Instead, it might be linked to damage within the sympathetic nerve circuit. Anti-NMDAR antibodies are thought to affect both the output of the vagus nerve in the brainstem and the regulation of sympathetic nerve output in the hypothalamus, thus impacting the function of both the vagus and sympathetic nerves and leading to symptoms of autonomic nervous dysfunction. Experimental studies have also shed light on the potential mechanisms. For instance, injecting N-methyl-D-aspartate into the paraventral nucleus of the hypothalamus in rats has been shown to induce sympathetic excitation, increasing blood pressure and heart rate [28]. Conversely, administering ketamine, a non-competitive antagonist of NMDAR, can cause autonomic dysfunction [29], suggesting a direct relationship between NMDA receptors and autonomic nervous function. Makoto Yamakawa et al. [30]. identified autoimmune encephalitis patients (mostly Anti-NMDAR encephalitis) with autonomic symptoms were positive for anti-α3 subunit of the ganglionic-type nicotinic acetylcholine receptor (gAChRα3) antibody in blood. The antibody may destroy α3β4 nicotinic AChRs in both autonomic ganglia and brain. However they could not verify whether anti-gAChR Antibodies are causative antibodies for autonomic dysfunction in autoimmune encephalitis. Although the precise mechanisms underlying autonomic dysfunction in anti-NMDAR encephalitis are yet to be fully understood, these studies indicate that anti-NMDAR encephalitis may damaged the center autonomic nervous network and peripheral nervous system, resulting in the loss of balance of sympathetic and parasympathetic functions.In this study, through multivariate logistic regression analysis, only the highest mRS score was identified as an independent risk factor for autonomic nervous dysfunction, while the modified mRS score served as a method for evaluating neurological function in patients. The presence of autonomic nervous dysfunction could lead to an increased mRS score, suggesting a reciprocal causal relationship between the two. The absence of other related independent risk factors in this analysis is thought to be due to the small sample size of the study.

Children with autonomic dysfunction exhibited a poorer prognosis, longer hospital stays, and higher hospitalization costs, aligning with findings from previous studies. Autonomic dysfunction has been identified as a risk factor for a poor prognosis in anti-NMDAR encephalitis [31]. This correlation may be due to a range of factors; autonomic dysfunction can lead to hypopnea and increased salivation, which are linked to higher rates of pulmonary infections and mechanical ventilation, all of which significantly impact prognosis. In this study, all children with autonomic dysfunction were initiated on second-line immunotherapy, reflecting the severity and treatment complexity associated with their condition. Additionally, the cerebrospinal fluid protein levels were higher in the group with autonomic dysfunction than in those without, suggesting more severe neuronal damage in the former group. Despite these differences, there were no significant disparities in the number of white blood cells in the cerebrospinal fluid, head magnetic resonance imaging abnormalities, or anti-NMDAR antibody titers in both blood and cerebrospinal fluid between the two groups. This indicates that while autonomic dysfunction significantly affects clinical outcomes, it may not be directly reflected in these specific biomarkers.

The treatment of autonomic nervous dysfunction in anti-NMDAR encephalitis primarily involves symptomatic management. Since the clinical manifestations of hypertension, tachycardia and profuse hyperhidrosis are caused by sympathetic overactive, sympatholytic and parasympathomimetic medications such as beta-blockers, alpha-2 agonists, or acetylcholinesterase inhibitors may be used. Parasympathetic overactivity accounts for atrioventricular block, sinus bradycardia and cardiac arrest, so parasympatholytic (e.g. atropine),beta-2 agonists(e.g. isoprenaline) and temporary pacemakers should be used. Because central hypopnea which will not disappear quickly, which can lead to apnea and cardiovascular events, tracheal intubation or tracheotomy should be performed promptly, followed by mechanical assisted ventilation.Due to the the special nature of pediatric patients, there may be some risks when pediatric patients take these medications, but there is a lack of relevant research.Notably, symptoms related to autonomic dysfunction often resolve following immunotherapy.

## Conclusions

In summary, over one-third of children with anti-NMDAR encephalitis experience autonomic nervous dysfunction, with cardiovascular autonomic dysfunction being the most common form. This dysfunction not only exacerbates the severity of anti-NMDAR encephalitis but can also lead to life-threatening conditions such as cardiac arrest. Therefore, it is crucial to monitor for the onset of autonomic symptoms closely. Children with anti-NMDAR encephalitis and autonomic dysfunction often have a high incidence of consciousness disorders, prolonged duration of these disorders, poor prognosis, and extended hospital stays, underscoring the necessity of promptly initiating second-line immunotherapy.Early recognition, aggressively cardiorespiratory support in the Intensive Care Unit and supportive care treatment of autonomic dysfunction can improve the prognosis of pediatric patients with anti-NMDA receptor encephalitis. Healthcare providers should also recognize and treat autonomic dysfunction manifestations of pediatric patients with anti-NMDA receptor encephalitis in time.

Due to insufficient power from this small case–control clinical study, our results presented here should be regarded as preliminary. It was also a retrospective single center study, which might have resulted in selection bias, lake of external validation. Our study lays a foundation for future studies. We hope to have prospective, multicenter, observational and a larger sample size study to sasaaanalyzed the risk factors of autonomic dysfunction in Anti-NMDAR encephalitis pediatric patients in the future.

## Supplementary Information

Supplementary Material 1.

## Data Availability

The datasets used and/or analysed during the current study are available from the corresponding author on reasonable request.

## Abbreviations

NMDAR: Anti-N-methyl-D-aspartic receptor
GCS: Glasgow Coma Score
mRS: The modified Rankin Scale
CSF: Cerebrospinal flfluid
MRI: Magnetic resonance imaging
WBC: White blood cell count
gAChRα3: Anti-α3 subunit of the ganglionic-type nicotinic acetylcholine receptor

## References

[1] Ren H, Fan S, Zhao Y, Guan H. The changing spectrum of antibody-mediated encephalitis in China. Neuroimmunol. 2021. 10.1016/j.jneuroim.2021.577753. 10.1016/j.jneuroim.2021.577753 34739913

[2] Varley JA, Webb AJS, Balint B, et al. The movement disorder associated with NMDAR antibody-encephalitis is complex and characteristic:an expert video-rating study. J Neurology. 2019. 10.1136/jnnp-2018-318584. 10.1136/jnnp-2018-318584 PMC6581096 30032119

[3] Hu CC, Pan XL, Zhang MX, Chen HF. Paroxysmal speech disorder as the initial symptom in a young adult with anti-N-methyl-D-aspartate receptor encephalitis: A case report. World J Clin Cases. 2022. 10.12998/wjcc.v10.i24.8648. 10.12998/wjcc.v10.i24.8648 PMC9453376 36157799

[4] Hébert J, El-Sadi F, Maurice C, Wennberg RA, Tang-Wai DF. Adult-Onset Anti-N-methyl-D-aspartate-receptor Encephalitis Presenting as a Non-Fluent Aphasia. Can J Neurol Sci. 2018. 10.1017/cjn.2017.280. 29249215 10.1017/cjn.2017.280

[5] Graus F, Titulaer MJ, Balu R, et al. A clinical approach to diagnosis of autoimmune encephalitis. Lancet Neurol. 2016. 10.1016/S1474-4422(15)00401-9. 26906964 10.1016/S1474-4422(15)00401-9 PMC5066574

[6] Baysal-Kirac L, Tuzun E, Erdag E, et al. Neuronal autoantibodies in epilepsy patients with peri-ictal autonomic findings. J Neurol. 2016. 10.1007/s00415-015-8002-2. 26725084 10.1007/s00415-015-8002-2

[7] von Elm E, Altman DG, Egger M, et al. The Strengthening the Reporting of Observational Studies in Epidemiology (STROBE) statement: guidelines for reporting observational studies. J Clin Epidemiol. 2008. 10.1016/j.jclinepi.2007.11.008. 10.1016/j.jclinepi.2007.11.008 18313558

[8] Yan L, Zhang S, Huang X, Tang Y, Wu J. Clinical Study of Autonomic Dysfunction in Patients With Anti-NMDA Receptor Encephalitis. Front Neurol. 2021. 10.3389/fneur.2021.609750. 33613429 10.3389/fneur.2021.609750 PMC7894204

[9] Titulaer MJ, McCracken L, Gabilondo I, et al. Treatment and prognostic factors for long-term outcome in patients with anti-NMDA receptor encephalitis:an observational cohort study. Lancet Neurol. 2013. 10.1016/S1474-4422(12)70310-1. 23290630 10.1016/S1474-4422(12)70310-1 PMC3563251

[10] Byun JI, Lee ST, Moon J, et al. Cardiac sympathetic dysfunction in anti-NMDA receptor encephalitis. Auton Neurosci. 2015. 10.1016/j.autneu.2015.08.002. 26275576 10.1016/j.autneu.2015.08.002

[11] Dalmau J, Gleichman AJ, Hughes EG, et al. Anti-NMDA-receptor encephalitis: case series and analysis of the effects of antibodies. Lancet Neurol. 2008. 10.1016/S1474-4422(08)70224-2. 18851928 10.1016/S1474-4422(08)70224-2 PMC2607118

[12] Chen Z, Zhang Y, Wu X, Huang H, Chen W, Su Y. Characteristics and Outcomes of Paroxysmal Sympathetic Hyperactivity in Anti-NMDAR Encephalitis. Front Immunol. 2022. 10.3389/fimmu.2022.858450. 35464412 10.3389/fimmu.2022.858450 PMC9020260

[13] Lee M, Lawn N, Prentice D, Chan J. Anti-NMDA receptor encephalitis associated with ictal asystole. J Clin Neurosci. 2011. 10.1016/j.jocn.2011.03.024. 21992741 10.1016/j.jocn.2011.03.024

[14] Nazif TM, Vázquez J, Honig LS, Dizon JM. Anti-N-methyl-D-aspartate receptor encephalitis: an emerging cause of centrally mediated sinus node dysfunction. Europace. 2012. 10.1093/europace/eus014. 22345374 10.1093/europace/eus014 PMC3404556

[15] Kawabe T, Chitravanshi VC, Nakamura T, Kawabe K, Sapru HN. Mechanism of heart rate responses elicited by chemical stimulation of the hypothalamic paraventricular nucleus in the rat. Brain Res. 2009. 10.1016/j.brainres.2008.10.059. 19022229 10.1016/j.brainres.2008.10.059 PMC2649118

[16] Wang GL, Yin F, Wang Y, et al. Zhonghua Er Ke Za Zhi. 2019. 10.3760/cma.j.issn.0578-1310.2019.02.012.

[17] Florance NR, Davis RL, Lam C, et al. Anti-N-methyl-D-aspartate receptor (NMDAR) encephalitis in children and adolescents. Ann Neurol. 2009. 10.1002/ana.21756. 19670433 10.1002/ana.21756 PMC2826225

[18] Wang Y, Zhang W, Yin J, et al. Anti-N-methyl-d-aspartate receptor encephalitis in children of Central South China: Clinical features, treatment, influencing factors, and outcomes. J Neuroimmunol. 2017. 10.1016/j.jneuroim.2017.09.005. 28935354 10.1016/j.jneuroim.2017.09.005

[19] Dou X, Li D, Wu F, et al. The clinical features, treatment and outcomes of 33 children from Northwestern China with Anti-N-methyl-D-aspartate receptor encephalitis. Neurol Res. 2022. 10.1080/01616412.2021.2000824. 34806564 10.1080/01616412.2021.2000824

[20] Li XJ. Clinical features of autoimmune encephalitis in children. Chinese Journal of Practical Pediatrics Clinical. 2019. 10.3760/cma.j.issn.2095-428X.2019.24.002.

[21] de Montmollin E, Demeret S, Brulé N, et al. Anti-N-Methyl-d-Aspartate Receptor Encephalitis in Adult Patients Requiring Intensive Care. Am J Respir Crit Care Med. 2017. 10.1164/rccm.201603-0507OC. 27552490 10.1164/rccm.201603-0507OC

[22] Benarroch EE. Physiology and Pathophysiology of the Autonomic Nervous System. Continuum (Minneap Minn). 2020. 10.1212/CON.0000000000000817. 31996619 10.1212/CON.0000000000000817

[23] Gibbons CH. Basics of autonomic nervous system function. Handb Clin Neurol. 2019;160:407–18. 10.1016/B978-0-444-64032-1.00027-8. 31277865 10.1016/B978-0-444-64032-1.00027-8

[24] Sposato LA, Hilz MJ, Aspberg S, et al. Post-Stroke Cardiovascular Complications and Neurogenic Cardiac Injury: JACC State-of-the-Art Review. J Am Coll Cardiol. 2020. 10.1016/j.jacc.2020.10.009. 33272372 10.1016/j.jacc.2020.10.009

[25] McDougall SJ, Münzberg H, Derbenev AV, Zsombok A. Central control of autonomic functions in health and disease. Front Neurosci. 2015. 10.3389/fnins.2014.00440. 25620907 10.3389/fnins.2014.00440 PMC4288135

[26] Dampney RA. Central neural control of the cardiovascular system: current perspectives. Adv Physiol Educ. 2016. 10.1152/advan.00027.2016. 27445275 10.1152/advan.00027.2016

[27] Fontes MAP, Filho ML, Santos Machado NL, et al. Asymmetric sympathetic output: The dorsomedial hypothalamus as a potential link between emotional stress and cardiac arrhythmias. Auton Neurosci. 2017. 10.1016/j.autneu.2017.01.001. 28131565 10.1016/j.autneu.2017.01.001

[28] Kawabe T, Chitravanshi VC, Kawabe K, Sapru HN. Cardiovascular function of a glutamatergic projection from the hypothalamic paraventricular nucleus to the nucleus tractus solitarius in the rat. Neuroscience. 2008. 10.1016/j.neuroscience.2008.02.076. 10.1016/j.neuroscience.2008.02.076 PMC2481515 18424005

[29] Krystal JH, Karper LP, Seibyl JP, et al. Subanesthetic effects of the noncompetitive NMDA antagonist, ketamine, in humans. Psychotomimetic, perceptual, cognitive, and neuroendocrine responses. Arch Gen Psychiatry. 1994. 10.1001/archpsyc.1994.03950030035004. 10.1001/archpsyc.1994.03950030035004 8122957

[30] Yamakawa M, Mukaino A, Kimura A, et al. Antibodies to the α3 subunit of the ganglionic-type nicotinic acetylcholine receptors in patients with autoimmune encephalitis. J Neuroimmunol. 2020. 10.1016/j.jneuroim.2020.577399. 32980672 10.1016/j.jneuroim.2020.577399

[31] Zhang Y, Liu G, Jiang M, Chen W, He Y, Su Y. Clinical Characteristics and Prognosis of Severe Anti-N-methyl-D-aspartate Receptor Encephalitis Patients. Neurocrit Care. 2018. 10.1007/s12028-018-0536-6. 29651625 10.1007/s12028-018-0536-6

